# Helminthiasis among School-Age Children and Hygiene Conditions of Selected Schools in Lafia, Nasarawa State, Nigeria

**DOI:** 10.3390/tropicalmed4030112

**Published:** 2019-07-29

**Authors:** Eniola M. Abe, Onyinye C. Echeta, Akwashiki Ombugadu, Linus Ajah, Peter O. Aimankhu, Akinola S. Oluwole

**Affiliations:** 1 National Institute of Parasitic Diseases (NIPD), Chinese Centre for Disease Control and Prevention, World Health Organization Collaborating Centre for Tropical Diseases, Shanghai 200025, China; 2 Department of Zoology, Federal University of Lafia, P.M.B 146, Lafia 950101, Nigeria; 3 Department of Pure and Applied Zoology, Federal University of Agriculture Abeokuta, P.M.B 2240, Abeokuta 110124, Nigeria

**Keywords:** helminthiasis, school-age, hygiene, schools, Lafia, Nigeria

## Abstract

The burden of soil-transmitted helminths (STHs) infections in Nigeria is enormous with serious public health significance. This study, therefore, assessed helminthiasis among school-age children and the hygiene conditions of schools in Lafia, Nasarawa State, Nigeria between December 2015 and April 2016 from four randomly selected primary schools. Stool samples were collected from 200 primary school pupils including 80 males (40%) and 120 females (60%) between five and 16 years, using clean sample bottles and a standard parasitology examination technique at the central laboratory at the Federal University, Lafia. An overall prevalence of 33.5% (67/200) helminths infections was recorded. A checklist of *Ascaris lumbricoides*, hookworm, *Trichuris trichiura*, and *Strongyloides stercoralis* was generated from the pooled data of the four studied schools in which *A. lumbricoides* occurred highest with 13% (26/200) while *S. stercoralis* was the least prevalent at 2.50% (5/200). Among the schools sampled, St. James Pilot Science Primary School’s children were the most infected at 44% (22/50). Multiple infections were observed in three of the four schools sampled. There was no significant difference (*p* > 0.05) in prevalence rates of different STHs infections in relation to age group and gender across schools. Our findings showed that the hygiene conditions in the studied schools were poor without water, hand washing materials, refuse bins, as well as poor sanitary conditions. This study also identified ova and larvae of STHs parasites in the analyzed soil samples from the studied schools. Most school-age children had knowledge about contamination but few among them washed their hands with water and soap. The obtained result indicated a negative association between the prevalence of STHs and the proportion of pupils that cleaned up with water after defection. We, therefore, advise that hygiene conditions in schools be improved and that the government should prioritize enrolling all primary schools in Nasarawa state for the school health program so as to reduce the burden of STHs among school-age children in the state.

## 1. Introduction

Soil-transmitted helminths (STHs) are a group of parasitic nematodes that cause infection in humans through contact with parasite eggs (*Ascaris lumbricoides* and *Trichuris trichiura*) or larvae (hookworm and *Strongyloides stercoralis*). They thrive in the warm and moist soil of the world’s tropical and subtropical countries [1]. STHs infections belong to the Neglected Tropical Diseases (NTDs) and their distribution is widespread globally [2,3]. The most recent estimates suggest that 819 million people worldwide are infected with *A. lumbricoides*, 465 million with *T. trichiura*, and 439 million with hookworm. Though neglected, *S. stercoralis* prevalence in the tropics and sub-tropics is significant with about 100 million people infected but with no disability adjusted life year (DALY) burden estimates [1,3,4].

The high prevalence of STHs have been associated with poverty, poor environmental hygiene, lack of adequate facilities for water supply and sanitation, ignorance, and impoverished health services [5,6]. 

STHs are one of the NTDs targeted for elimination by 2020 [7]. The London 2012 declaration fueled commitment towards the elimination of NTDs as a public health problem globally, particularly the preventive chemotherapy NTDs. Currently albendazole/mebendazole is the drug of choice for mass treatment in highly endemic areas. However, studies have shown that the prevalence of STHs return to its initial level six months after treatment [8] due to re-infection, especially in areas where indiscriminate defecation, poor personal hygiene, lack of adequate sanitary facilities, and water supply is prevalent. Hence, the World Health Organization (WHO) has recommended the provision of water, sanitary facilities, and good hygiene practice in endemic communities as complementary to chemotherapy in order to achieve the elimination goal. 

The persistent burden of STHs is mostly reported in children living in developing countries with the prevalence rate ranging between 50% and 80% [9,10,11]. This is because of their activity and continuous exposure to contaminated soil and water [12,13]. Studies have indicated the prevalence of intestinal parasites in Nasarawa State, Nigeria [14,15]. However, there is a lack of information on the demography and hygiene conditions of schools in Nasarawa State. Such information is important as they will help guide the development of school health programs which are requirements for the sustainable control of soil-transmitted helminths in school children [16]. This study, therefore, aims to investigate the hygiene conditions in schools, evaluate demographic features, and intestinal helminth infections among school-age children and identify factors that are essential for the development of sustainable school health programs.

## 2. Materials and Methods

### 2.1. Study Area

This study was carried out in Lafia, the state capital of Nasarawa State. Nasarawa State is located in the north central region of Nigeria with a land area of 27,137.8 square kilometers. Nasarawa State lies within the guinea Savannah region and has a tropical climate with moderate rainfall (annual mean rainfall of 1311:75 cm) and shares borders with Kaduna State to the north, the Federal Capital Territory Abuja to the west, Plateau and Taraba States to the east, and with Benue and Kogi States to the south. The state has 13 local government areas including Awe, Nasarawa, Nasarawa-Eggon, Obi, Toto, Wamba, Akwanga, Doma, Karu, Keana, Keffi, Kokona, and Lafia with farming, trading, artisans, and civil servants being their major occupations and Hausa as the major spoken language.

### 2.2. Ethical Approval

Ethical approval to conduct the survey with reference number NS/UBEB/S/EDU-259/VOL.1 was approved on 19 November 2015 and obtained from the Nasarawa State Universal Basic Education Board alongside verbal consent from authorities of the four public schools used for the study. The parents or guardians of the school children were also informed about this study so as to seek their consent. Pupils whose parents did not sign the consent form were, however, not recruited into this study.

### 2.3. Selection of Schools

This study was carried out in four randomly selected public primary schools in Lafia, Nasarawa State namely Ta’al Model Primary School (Latitude 8.519037°; Longitude 8.521850°), Lafia East Pilot Science Primary School (8.492773°; 8.529467°), LGEA Mararaba (Latitude 8.474422°; Longitude 8.583168°), and St. James Pilot Science Primary School (Latitude 8.498988°; Longitude 8.513107°). The studied schools are at an average of 4000 m apart from each other. The schools were randomly selected in the study area following stratification to represent the predominant socioeconomic status of the location. We, however, could not assess private primary schools in this study because we were not given access, which informed our decision to use Ta’al Model Primary School as it has similar characteristics to an average private primary school in our study location.

### 2.4. Surveys and Sample Collection

#### 2.4.1. Collection and Examination of Stools Sample for Presence of STH 

Sterile plastic bottles with unique identification number were provided to the randomly selected school pupils (5–16 years) using the ballot system from the selected schools. The pupils were asked to take the sample bottles home and provide their early morning faeces in the sample bottles for parasitological examination. Using WHO guidelines [17], 10 students were randomly selected per class while 50 students were selected in each school. Demography information such as age, sex, and class were obtained from each child that participated in the study. Stool samples collected were taken to the central laboratory of the Federal University, Lafia. The stool samples were examined within 12 h using the cellophane thick smear method for intestinal helminthes eggs [18] and results were recorded on the corresponding pupil survey form. 

#### 2.4.2. Collection of Soil Samples in Selected Locations around the School Environment

Soil samples were collected at three different locations i.e. refuse sites, area close to defecation sites, and playgrounds or common sitting grounds of each school. Samples collected from each site were labelled A, B, and C respectively. At each site, 20 grams of soil samples was randomly collected at three different areas using a quadrant. The soil samples collected were processed using the sodium nitrate (NaNO_3_) floatation method and examined for the presence of helminth eggs/ova and worm at the central laboratory of the Federal University, Lafia. The eggs and larvae were identified based on morphological details as described by [19].

Each school’s sanitation conditions (specifically presence of toilet, type of toilet, availability of water, availability of hand washing soap) and the level of personal hygiene at home were also collected using an interviewer’s administered questionnaire.

#### 2.4.3. Questionnaire Administration

A total of 200 structured questionnaires were administered to the pupils to access information on their knowledge, attitude, and practice. 

### 2.5. Statistical Analysis

Data obtained were analyzed using R Console software (Version 2.9.2, R Foundation for Statistical Computing, Vienna, Austria). Proportions of infection rate of soil-transmitted helminths in stool samples as well as soil samples in the four selected schools were compared using Pearson’s Chi-square test. Descriptive statistics were used to measure risk factors, hygiene conditions, and mode of clean up among school-age children. Pearson’s product-moment correlation was used to compare the relationship between the prevalence of STHs and proportion of children that clean up with water after defecation. Statistical significance was measured at *p* < 0.05.

## 3. Results

### Summary of Infection Patterns

A total of 200 school children comprising of 80 (40%) boys and 120 (60%) girls attending the four public primary schools provided stool samples. STHs prevalence was highest among the 5–7 years age group with 42.6% prevalence and least in the 11–13 age group with 25.5% prevalence. STHs prevalence was highest in males with 36.3% while females had 31.7% (Table 1). However, the prevalence of infection among primary school children with respect to age and sex showed no significant difference (age: χ^2^ = 4.9605, df = 3, P = 0.1747; sex: χ^2^ = 0.27027, df = 1, P = 0.6032).

Out of the 200 fecal samples provided and screened, 67 (33.5%) were positive for STHs infections while those uninfected were 133 (66.5%). Therefore, the pooled proportions of uninfected pupils (66.5%) to those infected (33.5%) varied significantly (χ^2^ = 10.89, df = 1, P = 0.0009668). The checklist of STHs generated from the positive samples obtained from all four schools include, *A. lumbricoides* (13%), Hookworm (8%), *T. trichiura* (7.5%), and *S. stercoralis* (2.5%) (Table 2). St. James Pilot Science Primary School was implicated with the highest number of STHs infection of 22 (44.0%) among the sampled schools.

Multiple infections were observed in Lafia East Pilot Science Primary School, St. James Pilot Science Primary School and LGEA Mararaba but none was reported in Ta’al Model School (Table 2). 

**Figure 1:**
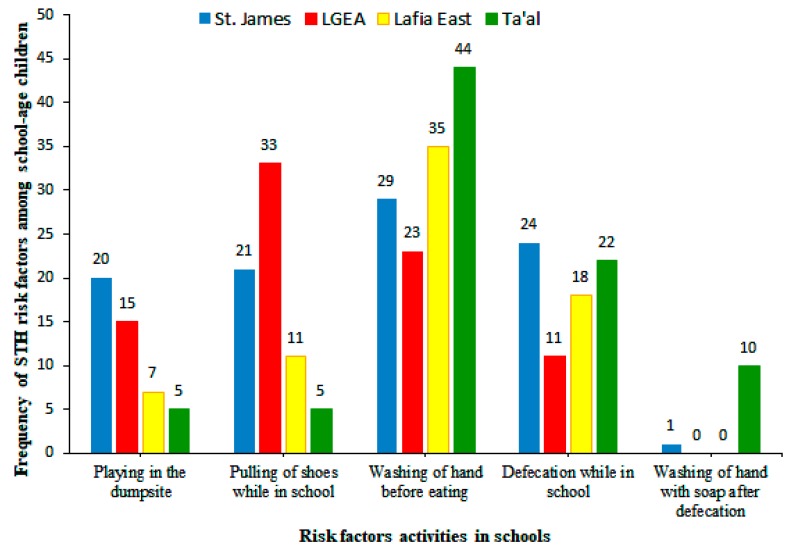
Factors that promote the transmission of soil-transmitted helminths among school-age children in relation to schools.

**Figure 2:**
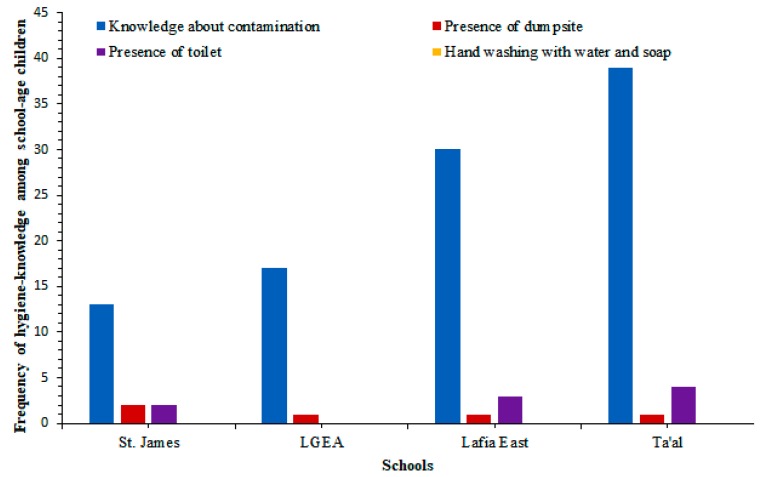
Knowledge about contamination and information on the presence of toilet facilities and hand-washing habits among school-age children.

Water supply was not available in three of the four schools sampled in this study. These include: Lafia East Pilot Science Primary School, LGEA Mararaba, and St. James Pilot Science Primary School while supply was not consistent in Ta’al Model Primary School. In addition, poor sanitary conditions, unavailability of hand washing soap, and the absence of refuse bins were observed as shown in Figure 1 and Figure 2.

**Figure 3:**
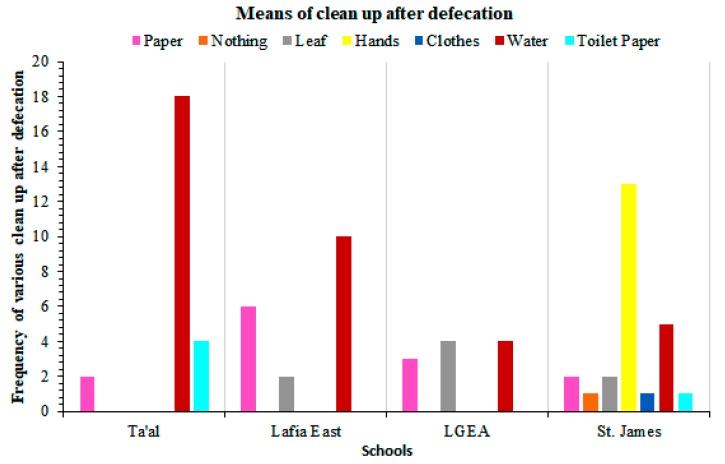
Mode of clean up after defecation among school-age children across schools.

Following the analysis of soil samples collected from different areas including toilet areas, playgrounds, and dumpsites within the four studied schools, *A. lumbriocoides* eggs, *T. trichiura* eggs, hookworm eggs, and one adult *A. lumbricoides* worm were identified. Furthermore, *Escherichia coli* and *Giardia lambdia* eggs were also identified from the analyzed soil samples (Table 3). Out of the four schools, soil samples from St. James Pilot Science Primary School had the highest number of parasitic soil helminthes while Ta’al Model Primary School recorded the lowest (Table 3). The mode of clean up after defecation is shown in Figure 3. A description of the hygiene condition indicators in the four schools is indicated in Table 4.

**Figure 4:**
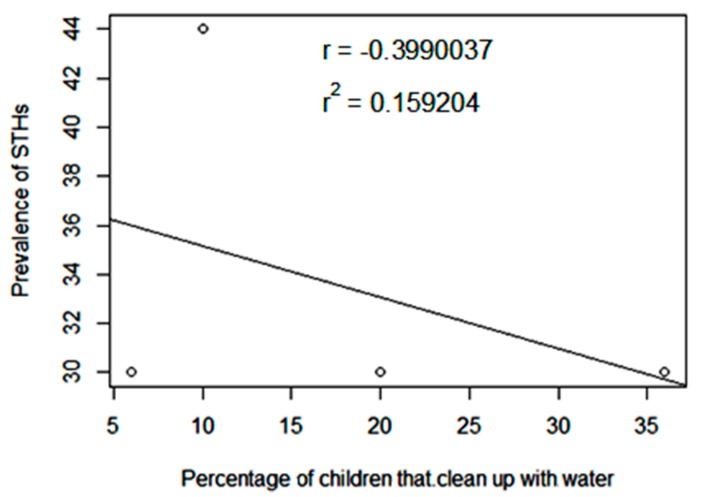
Association between prevalence of soil-transmitted helminths (STHs) and proportion of children that clean up with water.

Figure 4 shows a negative association between the prevalence of STHs and the proportion of children that clean up with water after defecation (t = −0.61538, df = 2, P = 0.601, r = −0.3990037, r^2^ = 0.159204). 

## 4. Discussion

Despite on-going intervention through the implementation of periodic mass deworming of pupils in the four selected schools, the burden of STHs is still high among school-age children. Findings from this study agree with Wagbasoma and Aisien [12] and Aniwada et al. [20] that recorded high STHs burden among children in Benin City and a rural community in Enugu State, Nigeria respectively.

This may possibly be due to the continuous exposure to sources of infection because of poor personal hygiene practice and environmental sanitation, lack of basic knowledge about the transmission of infections, as well as control and preventive measures against these infections. These pose serious threats to the wellbeing of most people living in the tropics [12,20].

An overall prevalence of 33.5% shows that STHs is of public health importance in Lafia, Nasarawa State, Nigeria. This value is more than the prevalence threshold set by WHO for yearly mass treatment of endemic communities. The major parasites found were *T. trichiuria*, *A. lumbricoides*, *S. stercoralis*, and hookworm, with *A. lumbricoides* being the most common STHs. 

Several studies have identified *A. lumbricoides* as the most common STHs infection globally [21,22,23,24]. Aniwada et al. [20] reported a similar outcome in Enugu with 25.6% of pupils found infected with STHs. Nduka et al. [25] also found a prevalence of 23.6% for STHs in Ishiagu, Abia State and reported that *A. lumbricoides* was the most prevalent with 34.7%, while others such as *T. trichiura*, hookworm and *Entamoeba histolytica* had 8.7%, 17.80%, and 14.80% respectively. A high prevalence of *A. lumbricoides* (52.7%) was also reported in a study conducted in Lusaka [26]. 

This study also reported 2.5% prevalence for multiple infection of *A. lumbricoides* and hookworm in three of the four schools sampled in Lafia. Nmorsi et al. [27] reported 35.57% prevalence of *A. lumbricoides* and hookworm multiple infection in the Akoko Edo area of Edo state. 

The high prevalence of *A. lumbricoides* may be due to the high resistance of the infective ova to desiccation and the direct mode of infection that enhances longevity and promotes infectivity [28]. On the other hand, children who play barefooted on contaminated grounds are at risk of infection with hookworm and *S. stercoralis*. Hence, the prevalence of hookworm and *S. stercoralis* among the pupils as observed in this study may be due to the pupil’s movement around the school environment, especially being barefooted on the playground during break time and after school hours. Such exposures in areas with soil contaminated with STHs allow for easy penetration of the skin by the parasites and initiation of infection.

Comparison of infection rates among the studied schools indicated no significance in the variation of STHs prevalence across age groups and sex which suggests that all ages and both genders are susceptible to STHs infection in the study area. Salawu and Ughele [29] reported that there was no variation in STHs prevalence in relation to age and sex among pupils in the Ife East local government area, Osun state, Nigeria. 

Adedayo and Akinlabi [30] and Uneke et al. [31] also showed that STHs infections among school children in a rural community in South-West Nigeria and a semi-urban area of South-Eastern Nigeria respectively have no gender preference. Meanwhile, Albonico et al. [32] and Naish et al. [33] indicated that STHs prevalence have no preferred age group. 

The pooled hygiene condition indicators in relation to the four selected schools suggests poor health provisions in the schools. Furthermore, the children were consistently being predisposed to environment contaminated with STHs parasites based on their unhygienic practices which promotes the survival and continuous transmission of these parasites. Grimes et al. [34] showed that poor hygiene level in schools, inadequate water supplies, and poor sanitary conditions contribute to the survival and continuous transmission of parasites, but access to potable water sources as well as adequate toilet facilities (water cistern) could help reduce the risk of STHs and other disease transmissions among pupils [11]. 

Although Ta’al Model Primary School has a water cistern, but poor access to water supply was a limitation and as such, the toilets were used by the pupils without flushing and washing their hands with soap after defecation; an action that could lead to the spread of parasites, especially the egg of *A. lumbricoides* which is known to thrive in all places [35].

Findings from our study indicated that about 36% of the pupils in Ta’al Model School clean up with water after defecation, followed by 20%, 10%, and 6% in Lafia East, St. James, and LGEA Primary Schools respectively. Hands not being properly washed with soap after defecation could be contaminated with STHs parasites, and consequently when they are used to eat or handle snacks, it could aid STHs transmission. Borghi et al. [36] demonstrated that handwashing habits with soap and water after defecation were very effective at reducing the burden of intestinal helminthic disease among children. 

Handwashing with soap and water after defecation should be encouraged to reduce transmission of STHs infections, especially *A. lumbricoides.* Cleaning up with water after defecation did not influence STHs prevalence. The level to which cleaning up with water after defecation influenced STHs prevalence in this study was 15.9% (coefficient of determination, r^2^ = 0.159204) while other factors may be responsible. These data are, however, preliminary because this association was based on a single data point (44% STH prevalence reported at St. James Pilot Science Primary School).

Our result showed that about 26% of the pupils clean up with their hands after defecation in St. James Primary School while this practice was not reported in the other three sampled schools. This practice might be responsible for the higher proportion of STHs infection rates reported in St. James’s Primary School. 

This study also indicated that 6% of pupils in Ta’al Model School defecate in the nearby bush while 18% of pupils in both St. James’s and LGEA Primary Schools indulge in a similar habit. The non-availability of refuse bins observed in all schools is also identified as a contributing factor to STHs transmission and other pathogens. Children were seen playing around the garbage, urinating, and even eating around the corner, which expose them to risk of infection with STHs. Refuse and waste sites are sources of various pathogens and are readily visited by houseflies, which are known vectors of disease pathogens and have the ability to transmit STHs [37].

The presence of STHs parasite eggs in all the soil samples analyzed clearly indicate poor hygiene conditions across the four schools. St. James’s School was implicated with the highest prevalence of *A. lumbricoides* (14%) among the four sampled schools. Defecation by pupils during the school period was found to be high in this school and the pupils did not wash their hands with washing soap after defecation. This action no doubt had an impact in promoting the transmission of infection in the school. 

Meanwhile, soil samples from all the sites in Ta’al Model School were infested with only *T. trichiura* eggs, unlike what was observed in the other schools. This could be attributed to better sanitation practices in the school compared to the other three schools. 

In addition, the pupils of Ta’al Primary School were found to exhibit a better level of personal hygiene when compared to those in St. James Primary School because 88% of pupils from Ta’al Primary School washed their hands before eating. Meanwhile, 44% of the population interviewed indicated that they defecated during school hours and 20% among them washed their hands with soap after defecation. 

Good sanitation and hygiene culture should be imbibed in order to promote the effective control of STHs among school-age children as well as society at large. The hygiene conditions of the pit latrines in the schools surveyed were bad and do not enhance healthy living, thus, the children preferred to indulge in open defecation, usually in surroundings close to the toilet areas, the refuse dumpsites, and nearby bushes. 

This study, however, lacks data on infection intensity and hopes to improve on this when the study is scaled up in the future. 

## 5. Conclusions

It is evident that the burden of STHs infections and poor sanitary conditions are of serious public health importance in the studied schools based on our findings. It is, therefore, imperative that hygiene conditions such as provision and access to potable water sources and the condition of sanitary facilities be improved in these schools. 

The government should prioritize enrolling all primary schools in Nasarawa state for school health programs including periodic deworming at least once a year [5], and also improve health education so as to constantly create awareness on the danger posed by these parasites, their mode of transmission, and the need for prevention against helminthic parasites to curtail the persistence of STHs transmission among school-age children in schools.

There is also a need to educate food vendors in schools to ensure that their food is prepared and served under hygienic conditions so that they would not serve as secondary transmitters of STHs infections.

## Figures and Tables

**Table 1:** Prevalence of soil-transmitted helminths (STHs) infection by age group and sex in the four selected public schools.

Ta’al Model School Lafia					Lafia East Pilot Science Primary School			LGEA Mararaba			St. James Pilot Science Primary School			Total No. Examined	Total No. Infected (%)	χ^2^	*P* Value
		No. Examined	No Infected with STHs	(%)	No. Examined	No Infected with STHs	(%)	No. Examined	No Infected with STHs	(%)	No. Examined	No Infected with STHs	(%)				
Age	5–7	9	0	0	11	6	54.5	17	6	35.3	10	8	80.0	47	20 (42.6)	4.9605 *	0.1747 *
	8–10	17	5	29.4	14	4	28.6	14	3	21.4	17	6	35.3	62	18 (29.0)		
	11–13	15	3	20	13	3	23.1	11	4	36.4	16	4	25.0	55	14 (25.5)		
	14–16	9	7	20	13	3	23.1	11	4	36.4	16	4	25.0	55	14 (25.5)		
Sex	Male	20	6	30.0	18	7	38.9	20	6	30.0	22	10	45.5	80	29 (36.3)	0.27027 **	0.6032 **
	Female	30	9	30.0	32	8	25.0	30	9	30.0	28	12	42.9	120	38 (31.7)		

* The output for comparison of the prevalence rate of STHs in relation to age groups. ** The output for comparison of the prevalence rate of STHs in relation to sex.

**Table 2:** Prevalence of soil-transmitted helminth parasites in the four selected public schools.

Parasites	Ta’al Model School			Lafia East Pilot Science Primary School			LGEA Mararaba			St. James Pilot Science Primary School			Total No. Examined	Total No. Infected	%	χ^2^	*P* Value
	No. Examined	No. Infected	%	No. Examined	No. Infected	%	No. Examined	No. Infected	%	No. Examined	No. Infected	%					
Any infection	50	15	30	50	15	30	50	15	30	50	22	44	200	67	33.5	3.2993	0.3477
*A. lumbricoides*	50	7	14	50	6	12	50	6	12	50	7	14	200	26	13.0	0.17683	0.9812
*T. trichiuria*	50	3	6	50	3	6	50	4	8	50	5	10	200	15	7.5	0.79279	0.8512
Hookworm	50	4	8	50	4	8	50	3	6	50	5	10	200	16	8.0	0.54348	0.9092
*S. stercoralis*	50	1	2	50	0	0	50	1	2	50	3	6	200	5	2.5	3.8974	0.2728
Multiple infection	50	0	0	50	2	4	50	1	2	50	2	4	200	5	2.5	2.2564	0.5209

**Table 3:** Prevalence of soil-transmitted helminths found in soil samples of the four selected public schools.

Site of Collection	Ta’al Model School			Lafia East Pilot Science Primary School			LGEA Mararaba			St. James Pilot Science Primary School		
	Toilet Area	Refuse Bin	Playground	Toilet Area	Refuse Bin	Playground	Toilet Area	Refuse Bin	Playground	Toilet Area	Refuse Bin	Playground
**Grams of soil taken**	60	60	60	60	60	60	60	60	60	60	60	60
**Grams examined**	20	20	20	20	20	20	20	20	20	20	20	20
**Nature**	None	12 *T. trichiura eggs*	None	35 *Ascaris lumbricoides* eggs; 1 adult *A. lumbricoides* worms	2 *T. trichiura eggs*	None	None	10 Hookworm eggs	5 *T. trichiura eggs*	5 *Ascaris lumbricoides* eggs	15 *Ascaris lumbricoides* eggs	6 *Ascaris lumbricoides* eggs; *Escherichia coli* and *Giardia lambdia eggs*
**Parasite found**												

**Table 4:** Description of the hygiene condition indicators in the four schools.

Indicators	Ta’al Model School Lafia	Lafia East Pilot Science Primary School	LGEA Mararaba	St. James Pilot Science Primary School
Condition of water supply	Not consistent	Not available	Not available	Not available
Type of toilet facility	Water cistern	Pit	None	Pit
Number of toilets	3	2	None	2
Adequacy of toilet facility	Not adequate	Not adequate	Not adequate	Not adequate
Condition of toilet	Clean	Dirty	Not applicable	Dirty
Usage of toilet	In use	Not always	No toilet	In use
Soap for hand washing after defecation	Absent	Absent	Absent	Absent
Garbage around school compound	Present	Present	Present	Present
Presence of bushes	No	Yes	Yes	Yes
Food vendors	Yes	Yes	Yes	Yes
